# *In Situ* Generation of Nanoparticles on and within Polymeric Materials

**DOI:** 10.3390/polym16111611

**Published:** 2024-06-06

**Authors:** Antonios Kelarakis

**Affiliations:** UCLan Research Centre for Smart Materials, School of Pharmacy and Biomedical Sciences, University of Central Lancashire, Preston PR1 2HE, UK; akelarakis@uclan.ac.uk

**Keywords:** polymer nanocomposites, carbon dots, metallic nanoparticles, antimicrobial, antifouling, *in situ* synthesis, melt extrusion

## Abstract

It is well-established that the structural, morphological and performance characteristics of nanoscale materials critically depend upon the dispersion state of the nanofillers that is, in turn, largely determined by the preparation protocol. In this report, we review synthetic strategies that capitalise on the *in situ* generation of nanoparticles on and within polymeric materials, an approach that relies on the chemical transformation of suitable precursors to functional nanoparticles synchronous with the build-up of the nanohybrid systems. This approach is distinctively different compared to standard preparation methods that exploit the dispersion of preformed nanoparticles within the macromolecular host and presents advantages in terms of time and cost effectiveness, environmental friendliness and the uniformity of the resulting composites. Notably, the *in situ*-generated nanoparticles tend to nucleate and grow on the active sites of the macromolecular chains, showing strong adhesion on the polymeric host. So far, this strategy has been explored in fabrics and membranes comprising metallic nanoparticles (silver, gold, platinum, copper, etc.) in relation to their antimicrobial and antifouling applications, while proof-of-concept demonstrations for carbon- and silica-based nanoparticles as well as titanium oxide-, layered double hydroxide-, hectorite-, lignin- and hydroxyapatite-based nanocomposites have been reported. The nanocomposites thus prepared are ideal candidates for a broad spectrum of applications such as water purification, environmental remediation, antimicrobial treatment, mechanical reinforcement, optical devices, etc.

## 1. Introduction

Polymer nanocomposites have found applications related to energy conversion and storage; aerospace; sensing and actuation; environmental remediation; national defense; electronics; biotechnology; agriculture; healthcare; textiles; food packaging; and corrosion prevention [1,2,3,4]. The incorporation of nanoparticles (NPs) in polymeric matrices and coatings dramatically modifies the polymer chain dynamics and imparts improvements in terms of mechanical, optical, electrical, antimicrobial and barrier properties; dimensional and thermal stability; and fire retardancy [5,6]. To a certain extent, those performance enhancements largely depend on the dispersion state of the NPs that, in turn, are governed by the preparation method of the nanohybrid system [7]. Suffice it to say that well-dispersed polymer nanocomposites show characteristics that are superior compared to their micro-sized analogues and oftentimes demonstrate properties that exceed those predicted from the rule of mixtures.

Typically, surface treatments of the pre-formed NPs are employed to enhance particle–matrix interfacial adhesion, which also has a profound impact on their roughness, surface charge, surface energy, wettability, catalytic activity, reactivity, cellular uptake and biocompatibility [8]. To that end, biomolecules, polymers, small organic particles and functional groups are covalently anchored on NPs, while polymer wrapping, electrostatic forces, hydrophobic interactions, absorption effects, hydrogen bridging, van der Waals bonding and pi stacking are utilised in non-covalent modifications [9,10].

Well-explored strategies for the fabrication of polymer nanocomposites are based on incorporating preformed NPs to polymeric matrixes via melt-mixing, solution blending or *in situ* polymerisation [11]. Direct melt compounding relies on blending polymers and NPs under high-speed shearing at temperatures above their softening/melting points. By design, the method eliminates the use of solvents, is versatile, is cost-effective and is readily compatible with standard industrial processing. For example, intercalated poly(ethylene terephthalate) (PET)/clay nanocomposites with low toxicity and improved water vapor and ultraviolet (UV) transmission properties were prepared via melt extrusion using a twin screw micro-compounder at 265 °C under flowing nitrogen [12]. Likewise, well dispersed, electrically conductive polyamide 12 (PA12)/graphene nanocomposites with a low percolation threshold of 0.3 vol % were prepared by melt mixing at 220 °C [13]. The incorporation of organoclay by means of coextrusion with a twin screw at 200 °C imparts optical transparency to an otherwise opaque mixture of isotactic polypropylene (iPP) and poly(ethylene oxide) (PEO) [14]. The main limitation of the method is that the components are exposed to high temperatures that might lead to degradation, thus compromising both the performance and appearance of the nanocomposite.

Solution blending is based on the dispersion of the components within a suitable solvent (or a mixture of solvents) that is eventually removed from the system via evaporation, lyophilisation, coprecipitation or filtration [15]. For example, modified carbon nanofibres (MCNFs) and an elastomeric ethylene/propylene (EP) random copolymer were dispersed in xylene at 120 °C and then precipitated using cold methanol, and the nanocomposite thus derived showed good load transfer in the filler/polymer interphase [16]. Similarly, poly(vinylidene fluoride-co-hexafluoropropylene) (PVDF-HFP) and nanoclays were dispersed in *N*,*N*-dimethyl formamide (DMF), and the system was coagulated by adding water as an antisolvent; the resulting nanocomposites showed a high content of β phase and demonstrated improved elongation at break and an order of magnitude higher dielectric permittivity compared to their clay-free counterparts [17]. The drawbacks of this method are the use of toxic solvents, its tedious nature and the non-uniform distribution of NPs when solvent removal takes place over a prolonged period of time.

During *in situ* polymerisation, NPs are dispersed in a monomer-rich phase, and the growth of the polymer chains proceeds via standard reaction schemes [18]. For example, the *in situ* polymerisation of ε-caprolactam in the presence of multi-walled carbon nanotubes (MWCNT) led to well-dispersed nylon 6/MWCNT nanocomposites with an enhanced storage modulus (E′) and glass transition temperature (Tg) [19]. Similarly, poly(vinyl alcohol) (PVA)/graphene oxide (GO) nanocomposites with improved Young’s modulus and a dramatically reduced coefficient of moisture permeability were prepared via *in situ* free radical polymerisation of vinyl acetate in the presence of GO followed by alcoholysis [20]. Exfoliated clay/polystyrene (PS) nanocomposites were prepared via the free radical polymerisation of styrene in the presence of nanoclay layers that hosted the initiator in the intergallery spacing [21]. Issues related to the increasing viscosity of the growing macromolecules combined with the challenges of accurately controlling the polymerisation in the presence of NPs impose limits on the popularity of this method.

In this report, we review strategies that focus on the *in situ* growth of NPs within a polymer matrix, an approach conceptually different compared to the incorporation of pre-formed NPs to the host medium. By comparison, this method has been demonstrated for a rather limited number of systems where the growth of NPs can be triggered via radiation, a thermal event or a chemical reaction. The *in situ*-generated NPs are not simply kinetically trapped and passively immobilised within the matrix, but they tend to nucleate on the active sites of the macromolecular host, thus securing a high level of dispersion that is not prone to phase separation and NP agglomeration and migration.

## 2. Discussion

### 2.1. Metal NPs

#### 2.1.1. Role of Polymers to NP Growth

During the colloidal synthesis of metal NPs, different polymers such as poly(ethylene glycol) (PEG), poly(N-vinylpyrrolidone) (PVP), poly(vinylcaprolactame) (PVCL), PVA, cellulose, gellan, welan, pectin and κ-carrageenan can function as reducing agents, surface stabilisers, growth controllers and ligands [22]. For example, the nitrogen atoms and the carbonyl oxygen in PVP interact with metals, facilitating the synthesis of metallic and bimetallic NPs (Ag, Au, Pd, Pt, and their combinations), magnetite (Fe_3_O_4_), maghemite (γ-Fe_2_O_3_), hematite α-Fe_2_O_3_, ferrite CoFe_2_O, metal chalcogenide, metal telluride nanostructures, metal selenide, metal sulphated NPs and rare-earth oxides [23]. During the seed-mediated synthesis of Ag NPs, PVP effectively promotes the growth of specific facets, thus facilitating the formation of truncated cubes, truncated octahedrons, and octahedrons nanocrystals [24]. The reduction in Pt salt in the presence of poly(4-vinyl phenol) (PVPh) as both the reducing and the stabilizing agent gives rise to NPs with a size of 1.6–2.2 nm that show significant catalytic activity towards the borohydride reduction in p-nitrophenol and the hydrogenation of styrene and nitrobenzene in methanol [25].

#### 2.1.2. *In Situ* Deposition on Textiles Fibres

Ag NPs have been widely used in the textile industry due to their antimicrobial performance stemming from their ability to trigger oxidative stress to a range of strains, thus causing protein dysfunction and membrane damage [26]. The excessive and uncontrolled release of silver ions and NPs from textiles during laundering imposes health risks and constitutes a major environmental concern, necessitating the development of strategies that enhance the adhesion strength of silver NPs on fabrics [27,28].

**Figure 1:**
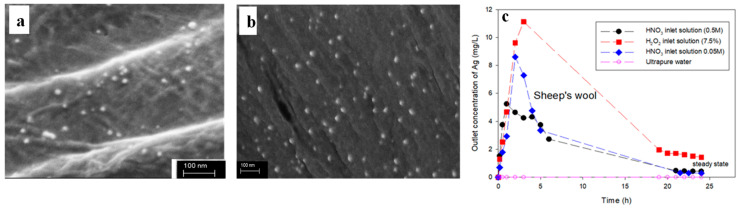
Scanning Electron Microscopy (SEM) images of (**a**) the surface and (**b**) the interior of bleached cotton fibres following the *in situ* formation of Ag NPs. (**c**) Outlet concentration of Ag as a function of time when *in situ*-modified wool fibres were subjected to flow-through experiments using the inlet solutions indicated (flow rate 1 mL/min). Adapted with permission from Ref. [29].

To this end, Montes-Hernandez et al. demonstrated that the *in situ*-generated Ag NPs can be irreversibly anchored to textile fibres (namely, untreated cotton, chemically beached cotton, sheep’s wool, polyamide (PA) and polyester) that are initially impregnated in AgNO_3_ and subsequently immersed in NaBH_4_ to allow for instantaneous silver reduction [29]. Interestingly, this process leads to the deposition of Ag NPs with sizes smaller than 20 nm not only on the surface (Figure 1a) but also at the interior of bleached cotton fibres (Figure 1b), an observation consistent with the significant activity against *Bacillus subtilis* of the *in situ*-modified fabric. Among the textiles tested, the wool fibres accommodated the highest number of Ag NPs (10 mg/g), followed by untreated cotton (2.3 mg/g), bleached cotton (1 mg/g), PA (0.6 mg/g) and polyester (0.3 mg/g). Moreover, the Ag NPs deposited on the wool fibres resist detachment when infused with ultrapure water, as shown in Figure 1c, due to the strong affinity of Ag ions with the carboxyl, hydroxyl and amino groups found on the surface of wool fibres. The oxidative resolution rates of Ag NPs reached a steady state following 20 h of continuous flow of H_2_O_2_ and HNO_3,_ and the low dissolution rates observed (Figure 1c) point to the strong adhesion of Ag NPs on the wool fibres.

**Figure 2:**
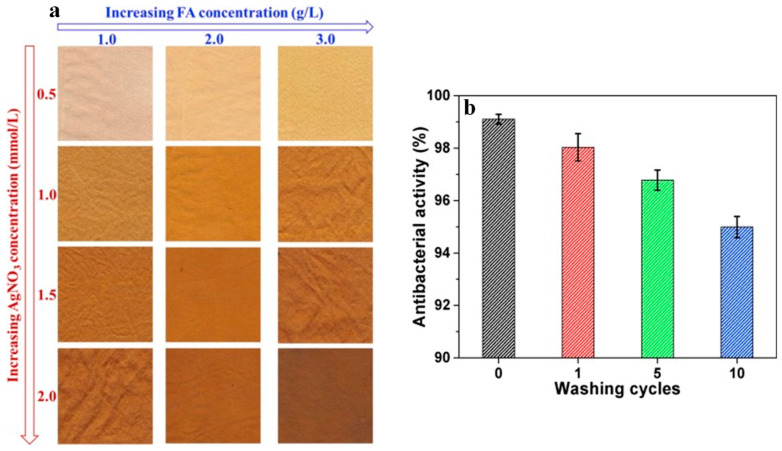
Silk fabric bearing *in situ*-generated Ag NPs: (**a**) their colour with respect to the AgNO_3_ and FA concentration; (**b**) their antibacterial activity against *E. coli* when subjected to a number of washing cycles. Adapted with permission from Ref. [33].

Likewise, cotton fabrics soaked in *Moringa oliefiera* leaf extract were immersed in aqueous CuSO_4_ solutions, and the nanocomposites prepared showed good antimicrobial properties against *Klebsiella pneumonia* and *Staphylococcus aureus* (*S. aureus*) [30]. A similar approach was used for the preparation of Ag/Cu bimetallic nanocomposite in cotton via reduction with *Aloe Vera* leaf extract [31]. Shahid et al. [32] reported the caffeic-acid-assisted reduction in AgNO_3_ to Ag NPs directly on the silk fabric surface. The silk fabrics show enhanced antimicrobial activity against *Escherichia coli* (*E. coli*), as is evident by the presence of a clear inhibition zone that is largely reserved after 10 washing cycles. Similarly, silk fabric was soaked with AgNO_3_ and was reduced with the natural polyphenol ferulic acid (FA), generating a family of nanocomposites that show a variety of colours from light cream/brown to dark golden/brown, depending on the concentration of the reactants (Figure 2a), while also demonstrating antimicrobial properties against *E. coli* that remain essentially unaltered after 10 washing cycles (Figure 2b) [33].

Likewise, *Pongamia pinnata* and *Tinospora cordifolia* leaf extracts were used as the reducing agents to generate Ag NPs on cellulose fabrics that showed antimicrobial activity [34,35]. In addition, Sadananda et al. [36] demonstrated the *in situ* generation of Cu NPs with an average diameter of 60 nm by immersing regenerated cellulose films firstly in *Ocimum sanctum* leaf extract and subsequently in aqueous CuSO_4_ solution. The nanocomposites show significant antimicrobial activity against *E. coli*, although the tensile properties and the thermal stabilities of the nanocomposites were compromised to a certain extent compared to neat cellulose.

**Figure 3:**
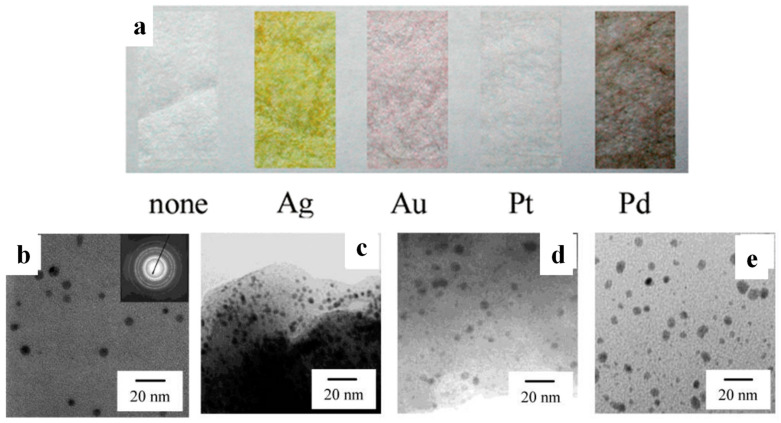
(**a**) Porous cellulose fibres bearing *in situ*-generated Ag, Au, Pt, and Pd NPs compared to the neat fibres. Transmission Electron Microscopy (TEM) images of porous cellulose fibres bearing *in situ*-generated Ag (**b**), Au (**c**), Pt (**d**), and Pd (**e**) NPs prepared from aqueous dispersions AgNO_3_, AuCl_3_, PtCl_4_ and Pd(NO_3_)_2_, respectively. Adapted with permission from Ref. [37].

Porous cellulose paper comprising uniform fibres (with an average diameter close to 11 μm) was immersed in aqueous dispersions of AgNO_3_, AuCl_3_, PtCl_4_ and Pd(NO_3_)_2_ followed by reduction via NaBH_4,_ giving rise to well-defined Ag, Au, Pt, and Pd NPs, respectively, tightly anchored on the surface and the amorphous part of the cellulose fibres (Figure 3). In contrast, only a negligible amount of Ag was deposited on non-porous starch and poly (vinyl alcohol) (PVA), highlighting the crucial role of the substrate’s structure and morphology in this process [37].

#### 2.1.3. *In Situ* Deposition on Polymeric Substrates

Silver NPs are oftentimes incorporated into different types of membranes (distillation, reverse osmosis, microfiltration and ultrafiltration) in order to impart antimicrobial and antifouling properties, aid in the photocatalytic degradation of pollutants, improve filtration efficiency, and mitigate pore blockage. Minimizing the leaching of the embedded Ag NPs to the filtrate and the environment remains a critical challenge in membrane technology [38].

**Figure 4:**
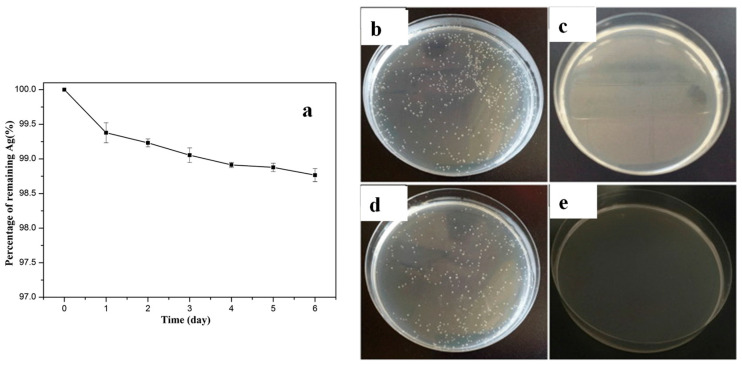
(**a**) Percentage of Ag NPs remaining immobilised on the surface of TA-Fe-PEI/Ag-modified membrane following its immersion in aqueous NaHCO_3_ with pH = 8.2. Photos of the Petri dishes containing *B. subtilis* (**b**,**c**) and *E. coli* (**d**,**e**) cultures exposed for 1.5 h to unmodified PA (**c**,**e**) and TA-Fe-PEI/Ag-modified membrane (**b**,**d**). Adapted with permission from Ref. [39].

Dong et al. reported that an ultrathin precursor layer of tannic acid-ferric ion-polyethylenimine (TA-Fe-PEI) was deposited on the PA reverse osmosis (RO) membrane and was subsequently immersed into a solution containing silver ammonia and polyvinylpyrrolidone (PVP), resulting in a TA-Fe-PEI/Ag-modified membrane [39]. The process resulted in uniformly distributed Ag NPs (as confirmed via SEM), 98.75% of which remained immobilised on the surface of the membrane following 6 days of immersion in aqueous NaHCO_3_ with pH = 8.2 (Figure 4a). Moreover, the mortality levels of *B. subtilis* and *E. coli* approached 100% when exposed for 1.5 h to the TA-Fe-PEI/Ag-modified membrane (Figure 4b,d). In addition, the modified membrane demonstrated simultaneous enhancements in water flux, salt rejection and antifouling performance.

**Figure 5:**
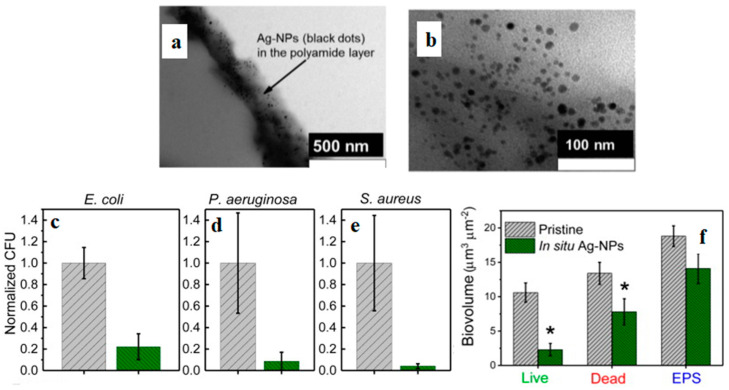
*In situ*-modified PA/Ag NPs membranes: (**a**,**b**) TEM images; reduction in the bacterial population of *E. coli* (**c**), *P. aeruginosa* (**d**) and *S. aureus* (**e**) exposed to *in situ*-modified membranes (green) compared to their pristine counterparts (grey). (**f**) Biovolumes of live, dead and extracellular polymeric substances (EPS) images of *P. aeruginosa* biofilm grown for 24 h on pristine and *in situ*-modified PA membranes. Asterisks represent significant (*p* < 0.05) difference between groups. Adapted with permission from Ref. [40].

Ben-Sasson et al. [40] focused on an RO membrane that was covered with AgNO_3_ solution for 10 min, followed by NaBH_4_ solution for 5 min to give rise to Ag NPs uniformly and strongly adhered on the PA selective layer of the membrane (Figure 5a,b). The surface roughness, zeta potential, hydrophilicity and salt selectivity of the treated membranes were not compromised, although a minor reduction in water permeability was observed. Following 5 h incubation with the *in situ*-modified RO membrane, the population of *E. coli*, *P. aeruginosa* and *S. aureus* bacteria was reduced by 78 ± 12, 91 ± 8% and 96 ± 2.2%, respectively (Figure 5c–e). At the same time, the biovolume on the *in situ*-modified RO membrane was suppressed by 73%, 38% and 25% for live, dead and extracellular polymeric substances (EPS), respectively (Figure 5f).

**Figure 6:**
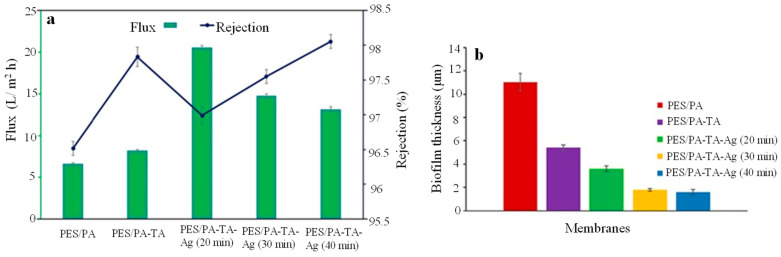
(**a**) Water flux and NaCl rejection of the *in situ*-modified PES/PA-TA-Ag membranes compared to their unmodified counterparts. (**b**) Average biofilm thickness deposited on PES/PA-TA-Ag membranes. Adapted with permission from Ref. [41].

Suresh et al. [41] demonstrated the build-up of highly effective desalination membranes starting from a commercial polyethersulfone (PES) membrane that was surface modified with a layer of PA generated via interfacial polymerisation (IP) reaction between aqueous m-phenylenediamine (MPD) and 1,3,5-benzenetricarbonyl trichloride (TMC), resulting in PES/PA. Consequently, PES/PA was grafted with TA for 1 min and cured for 30 s, leading to PES/PA-TA, and the *in situ* formation of Ag NPs within the network of TA took place via the impregnation of AgNO_3_ followed by reduction via NaBH_4_, while the reduction duration varied from 20 to 30 and 40 min. The water contact angle (WCA) of the membranes was found to be 68.2, 26.4, 18.6, 22.8, and 23.3° for PES/PA, PES/PA-TA, PES/PA-TA-Ag(20 min), PES/PA-TA-Ag(30 min) and PES/PA-TA Ag(40 min), respectively, an effect consistent with the large population of phenolic hydroxyl groups present in TA and the hydrophilic nature of Ag NPs. As shown in Figure 6a, the moderate concentration of Ag NPs in PES/PA-TA-Ag(20 min) improves water flux and decreases NaCl rejection, possibly due to pore formation and the development of amorphous areas on the PA layer, while the inclusion of higher concentrations of Ag NPs in PES/PA-TA-Ag(30 min) and PES/PA-TA-Ag(40 min) results in the opposite trends. As shown in Figure 6b, biofilm deposited on the PES/PA membrane had a thickness 11.05 ± 0.75 μm and was composed of living bacteria, while the biofilm deposited on PES/PA-TA-Ag(20 min) had a thickness 3.59 ± 0.24 μm was composed predominantly of dead bacteria.

Guo et al. [42] demonstrated that polydopamine (PDA) coating on the surface of reduced graphene oxide (RGO) can be prepared via the simultaneous reduction in GO with dopamine hydrochloride and the polymerisation of dopamine at pH = 8.5. A range of precursors, namely, AgNO_3_, H_2_PtCl_6_, solutions of Fe^2+^ and Fe^3+^, and titanium alkoxide are adsorbed on the PDA/RGO coating and are reduced *in situ* towards Ag/PDA/RGO, Pt/PDA/RGO, Fe_3_O_4_/PDA/RGO and TiO_2_/PDA/RGO, given that PDA coating functions as both the reducing and the capping agent for the nucleation, growth and stabilisation of NPs.

Esposti et al. [43] proposed the fabrication of porous poly(methyl methacrylate) (PMMA) membranes doped with *in situ* synthesised ZnO NPs, prepared via slow evaporation of PMMA/Zn(OAc)_2_ in DMF and subsequent thermal annealing at 110 °C for 48 h. The PMMA/ZnO nanocomposites exhibit the characteristic UV-tuneable wettability of ZnO NPs, in contrast to the WCA of the neat PMMA that remains unaffected by UV radiation.

**Figure 7:**
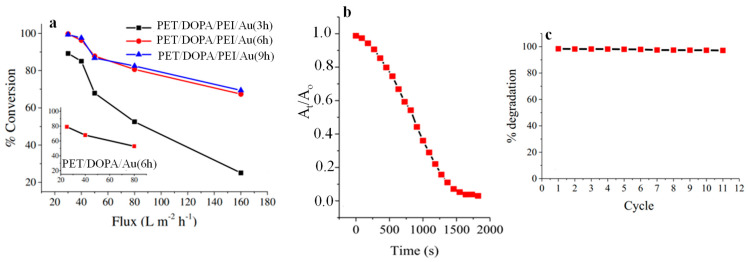
(**a**) PNP percental conversion in a flow membrane reactor with PET/DOPA/PEI/Au(3 h), PET/DOPA/PEI/Au(6 h) and PET/DOPA/PEI/Au(9 h) membranes as a function of flux. The inset displays data for PET/DOPA/Au(6 h). (**b**) Absorbance decrease at 495 nm during the degradation of Congo red under static conditions in the presence of PET/DOPA/PEI/Au(9 h) membrane using NaBH_4_ as the reducing agent as a function of time. (**c**) Degradation percentage of Congo red in a flow reactor with PET/DOPA/PEI/Au(9 h) over 11 cycles. Adapted with permission from Ref. [44].

Subair et al. [44] reported the fabrication of dopamine-coated PET track-etched microporous membranes PET/DOPA that were subsequently immersed in PEI to generate PET/DOPA/PEI. Both PET/DOPA and PET/DOPA/PEI were then dipped in an aqueous solution of HAuCl_4_ for different time intervals, and the *in situ* formation of Au NPs on both the surface and the pore walls took place on PET/DOPA in the absence of a reducing agent but in the presence of NaBH_4_ for PET/DOPA/PEI, yielding PET/DOPA/Au(xh) and PET/DOPA/PEI/Au(xh), respectively, where x stands for the number of hours allowed for the adsorption of [AuCl_4_]^−^ ions. To assess their catalytic activity, the membranes were mounted in a cell reactor and the reduction kinetics of p-nitrophenol (PNP) to p-aminophenol (PAP) (in a process where the embedded Au NPs facilitate electron transfer from the donor [BH_4_]^−^ ions to PNP) was monitored by UV spectroscopy (Figure 7a). More than a 60% reduction of PNP was recorded at high flow rates for PET/DOPA/PEI/Au(6 h) and PET/DOPA/PEI/Au(9 h) membranes, while more than a 99% reduction in PNP was recorded at a flow rate of 40 L m^−2^ h^−1^ due to longer residence time. The progress of Congo red degradation towards 1,1′-biphenyl sodium 4-amino-1-naphthalenesulfonate using NABH_4_ in the presence of the PET/DOPA/PEI/Au(9 h) membrane was monitored by absorbance measurements at 495 nm. The degradation kinetics are displayed in Figure 7b and indicate that the Au NPs were accessible and able to catalyze the reaction. When tested under flow conditions, the PET/DOPA/PEI/Au(9 h) membrane maintained more than 99% of its catalytic activity after 11 cycles of dye degradation (Figure 7c).

#### 2.1.4. *In Situ* Formation within a Polymer Matrix

An early study [45] demonstrated that ion-exchanging divinylbenzene-crosslinked sulfonated PS resigns with aqueous FeCl_2_ and FeCl_3_ results in the formation of optically transparent γ-Fe_2_O_3_/polymer nanocomposites with a saturation moment up to 46 emu/g and NP size 5–10 nm. Notably, when identical protocols were followed but in the absence of the polymer matrix, nonmagnetic micro-sized aggregates were formed. Those observations unambiguously demonstrate the critical role of the surrounding matrix in simultaneously providing active sites for NP nucleation; suppressing undesired aggregation; imposing constraints that limit the growth of NPs; altering the reaction mechanisms; promoting one crystalline phase at the expense of the others; and controlling the shape, size and morphology of the resultant NPs. In another study, the coprecipitation of ferrous (Fe^2+^) and ferric (Fe^3+^) ions (at basic conditions) in the presence of the thermosensitive poly(N-isopropylacrylamide-co-2-aminoethyl methacrylate) P(NIPAAm-co-AEM) resulted in the synthesis of magnetite (Fe_3_O_4_) polymer nanocomposites with the saturation of magnetisation 75 emu/g and significant DNA capture capabilities (up to 18.5 mg/g) [46].

Chosh et al. [47] demonstrated that *in situ*-generated Pt NPs, prepared via the thermal treatment of a solution-cast PVDF/H_2_PtCl_6_ film, preferentially stabilise the electroactive β and γ phase of the polymer at the expense of the inert α phase of the neat polymer, thus conferring ferroelectric polarisation, photoluminescence and piezoelectric properties

M. Mehrabanian et al. [48] reported the *in situ* formation of Pd and Pt NPs in chitosan films by UV pulsed laser irradiation by immersing chitosan films into precursor solutions of Na_2_PdCl_4_ or H_2_PtCl_6_- 6H_2_O, respectively, that were subsequently irradiated with Nd:YAG nanosecond laser. It was noted that prolonged irradiation promotes the fragmentation of the initially formed Pd NPs, while it favors agglomeration in Pt NPs.

Jeon et al. [49] reported the synthesis of PVP/Ag NPs nanocomposite films by dispersing the polymer and the Ag salt in N-methyl-2-pyrrolidinone followed by spin-coating on a glass substrate placed on a pre-heated aluminum plate. Ledo-Suarez et al. [50] demonstrated the *in situ* generation of 10 nm Au and Ag NPs by infusing the metal salt into amphiphilic epoxy gels derived via the reaction of a diepoxy monomer based on diglycidyl ether of bisphenol A with n-alkylamines. It was concluded that the secondary alcohols (that are oxidised to ketones) and tertiary amines in the polymeric backbone can effectively coordinate and reduce NP precursors. Pan et al. [51] prepared free standing chitosan (CS)/Ag NPs films that show antimicrobial activity against *E. coli* and *S. aureus* via electrooxidation of the silver electrode, given that the Ag^+^ ions are coordinated by the –COOH, –NH_2_ and –OH groups present in the carboxylated CS molecules and are being reduced by the –CH_2_OH groups. Mascia et al. [52] demonstrated the *in situ* synthesis of ZnO NPs in polyethyleneimine (PEI) by dispersing both polymer and Zn(C_5_H_7_O_2_)_2_ in ethanol followed by solvent evaporation. It was supported that the presence of polymer accelerated the nucleation and growth of ZnO NPs due to the basic environment created by the NH_2_ groups in the PEI.

**Figure 8:**
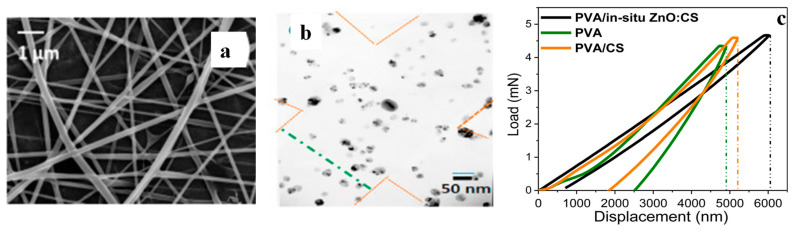
PVA/*in situ* ZnO:CS electrospun fibres (PVA/ZnO:CS ratio 40/60): (**a**) SEM image, (**b**) TEM image and (**c**) nanoindentation tests. In all cases, the applied voltage was 25 kV, the needle-tip collector distance was 20 cm, and the flow rate was 3 μL/s. Adapted with permission from Ref. [53].

CS was dispersed in CH₃COOH and was transferred into a Zn(CH_3_CO_2_)_2_·2H_2_O solution, to which NaOH was added dropwise; the system was maintained at 70 °C for 4 h, before the white solid ZnO:CS (comprising 77 wt% ZnO and 23 wt% CS) was collected [53]. The ZnO:CS was dispersed in CH₃COOH and mixed at different ratios with aqueous dispersions of PVA to ultimately generate bead-less electrospun fibres PVA/*in situ* ZnO:CS (Figure 8a) with a high level of dispersion of hexagonal ZnO NPs (Figure 8b). The nanocomposites exhibit significant antimicrobial activity against *S. aureus* and *E. coli*, while nanoindentation tests indicate that PVA and PVA/CS show a final penetration depth close to 2542 and 1853 nm, respectively, compared to only 716 nm for the PVA/*in situ* ZnO:CS (Figure 8c).

**Figure 9:**
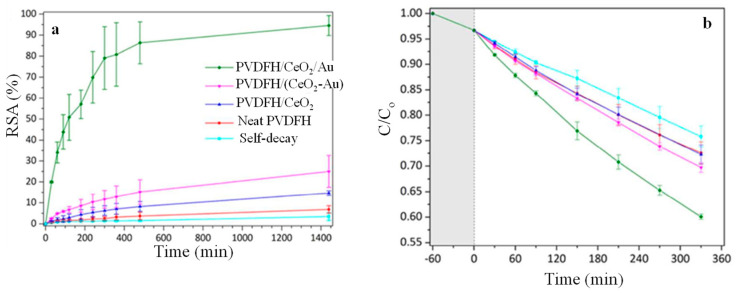
(**a**) Radical scavenging activity and (**b**) photocatalytic degradation activity against MB of the nanocomposites fibrous membranes as a function of time. Adapted with permission from Ref. [54].

Morselli et al. [54] reported the fabrication of poly(vinylidene fluoride-co-hexafluoropropylene) (PVDFH)/CeO_2_ fibrous membranes by electrospinning PVDFH/(NH_4_)_2_Ce(NO_3_)_6_ in DMF/acetone that was subsequently subjected to thermal annealing at 150 °C for 48 h. Furthermore, Au NPs were introduced to the system either by incorporating a gold salt along with cerium salt on the solution used for electrospinning or by dipping the PVDFH/CeO_2_ fibrous membranes on a gold precursor solution followed by thermal treatment, thus giving rise to PVDFH/(CeO_2_-Au) and PVDFH/CeO_2_/Au, respectively. The radical scavenging activity (RSA) of the nanocomposite fibrous membranes was determined by monitoring the decolouration of 2,2-Diphenyl-1-picrylhydrazyl (DPPH) in EtOH, as shown in Figure 9a. It can be clearly seen that the RSA of PVDFH/CeO_2_/Au approaches 90% within 24 h, indicating that the presence of Au NPs enhances the population of the active scavenging sites Ce^3+^ at the expense of Ce ^4+^. The photocatalytic degradation efficiency of the nanocomposite fibrous membranes against methylene blue (MB) was determined by UV-spectroscopy, as shown in Figure 9b. After 330 min of constant irradiation, the concentration of MB decreased by 30% for the PVDFH/(CeO_2_-Au) and by 40% for the PVDFH/CeO_2_/Au, which points to the narrowing of the bandgap of ceria induced by the Au NPs.

#### 2.1.5. *In Situ* Generation of Ag NPs via Melt Extrusion

**Figure 10:**
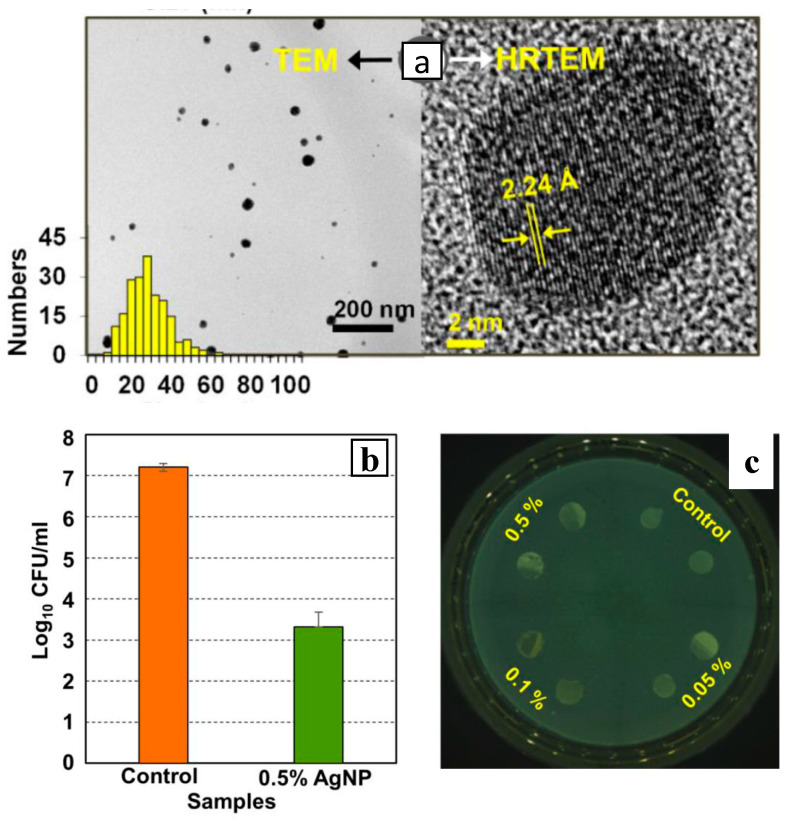
(**a**) TEM images (showing particle size histogram and lattice plane distance in Ag NPs) of the PA6-based nanocomposite prepared with 20 wt% Ag_2_O that was melt processed for 10 min at 240 °C. (**b**) Contact killing activity against *L. monocytogenes* of PA6 films containing 0.5 wt % of AgNP compared to the neat polymer (control). (**c**) Agar diffusion tests using PA6 nanocomposites with various Ag NPs loadings. Adapted with permission from Ref. [55].

Parida et al. [55] demonstrated a strategy that relies on the mild reducing capabilities of the thermoplastic polymer melts polyamide 6 (PA6), polypropylene (PP) and polylactic acid (PLA) to generate Ag NPs from their precursor salts Ag_2_O, Ag_2_CO_3_ and C_16_H_31_O_2_Ag during the melt extrusion process. This approach affords almost 100% conversion of the precursor salts dispersed in PA6 (at 245 °C) and PLA (at 165 °C) within the first 10 min of extrusion but only 60% conversion in PP (at 200 °C) due to the poor wetting of PP from the silver precursors. The method gives rise to well dispersed Ag NPs that show the characteristic d-spacing 2.23 Å of face-centred cubic (fcc) silver (Figure 10a), with a maximum loading of 18 wt%. The PA6/0.5wt% Ag NPs composite showed a 99.96% reduction in *Listeria monocytogenes* compared to the neat PA6 film (Figure 10b) and showed no inhibition zone (Figure 10c), suggesting that the Ag NPs resist detachment from the composite. With respect to the reduction mechanism, it has been suggested that during the initial stages of the extrusion the -CH_2_ and -NH_2_ groups in the PA6 melt create a reducing environment that facilitates the conversion of Ag_2_O to Ag^0^ and the reduction reaction enters its autocatalytic phase and is completed within 5 min at 240 °C. The activation energy of Ag_2_O thermal reduction is substantially reduced in the presence of PA6, without any detectable polymer degradation, yielding an homogeneous dispersion of Ag NPs with a wide size distribution around 20 nm.

In short, the *in situ* generation and growth of metallic and bimetallic NPs (Ag, Au, Pt, Pd, ZnO, Cu and CeO_2_) on the surface of membranes and within polymeric matrices has been studied systematically for applications related to antimicrobial textiles and antifouling membranes with improved water flux and salt rejection. Although the adhesion of NPs to the polymeric matrix appears stronger compared to the *ex situ* counterparts, the long-term performance of those materials has yet to be reported.

### 2.2. Carbon-Based NPs

#### 2.2.1. Carbon Nanotubes (CNTs) and Graphene Oxide (GO)

**Figure 11:**
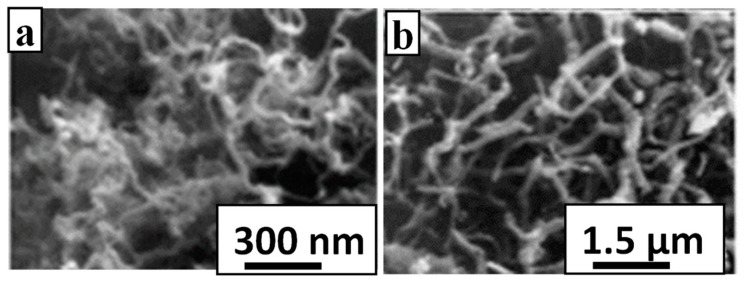
SEM images for CNT grown *in situ* on (**a**) Teflon and (**b**) polycarbonate. Adapted with permission from Ref. [59].

Carbon nanotubes and graphene-based polymer nanomaterials exhibit a series of attractive characteristics related to their mechanical, electrical, thermal, sensing and antimicrobial properties [56,57,58], although achieving a high level of dispersion is often challenging. Hong et al. [59] demonstrated the *in situ* growth of CNT on Teflon and polycarbonate (PC) when the polymeric substrates were painted with the catalyst cobalt naphthenate and a mixture of C_2_H_2_ (key building block) and H_2_S (sulphur has a promoter role via softening the metal catalyst) was introduced under microwave irradiation. The strategy affords curly CNTs on Teflon and short and straight CNTs on PC as seen in Figure 11.

Zaman et al. [60] reported a modified Hummers’ method for the *in situ* synthesis of GO within microcrystalline cellulose (MCC) derived from jute fibres. For this purpose, graphite flakes (G) and NaNO_3_ were dispersed in H_2_SO_4_ before KMnO_4_ was added to facilitate the generation of GO, followed by the addition of MCC and the temperature was raised gradually up to 95 °C, and the dark yellow slurry formed was retrieved via centrifugation. Hydrogen bonding is formed between the –OH groups of cellulose and the negatively charged groups on the edges of GO. The MCC/GO nanocomposite showed superior adsorptive capacity removing 98% of methylene blue within 135 min, demonstrating a maximum adsorption capacity of 751.9 mg/g.

#### 2.2.2. Carbon Dots (C-Dots)

C-dots represent a new type of fluorescent NPs typically comprising C, H, O and N that exhibit excitation wavelength emission with high quantum yields (QY) and minimal photobleaching. They are explored in applications related to sensing, drug delivery, bioimaging, forensics, agriculture, energy saving and storage, light emitting diodes and photocatalysis. Well-defined C-dots can be synthesised via cost- effective approaches that rely on the pyrolytic treatment of carbon rich precursors [61] such as mixtures of ethanolamine (EA) and citric acid (CA), urea and CA [62], biomass [63], etc. Alternatively, they are formed following top-down methods that rely on the decomposition of carbon structures such as CNTs, graphene- type materials, carbon fibres, carbon black via oxidation, electrooxidation, laser ablation and arc discharge.

**Figure 12:**
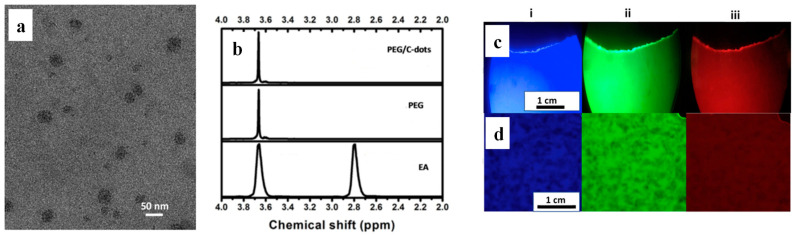
(**a**) TEM image of the C-dots extracted from PEG/C-dot nanocomposite. (**b**) ^1^H NMR spectra of PEG/C-dot, PEG and EA in D_2_O. Fluorescence microscopy images of (**c**) *in situ* generated PP/C-dot and (**d**) PEG/C-dot nanocomposite under (i) UV violet, (ii) blue and (iii) green excitation wavelength. Adapted with permission from Ref. [64].

Fernandes et al. [64] demonstrated that EA dispersed in polyethylene (PE), PP and polyethylene glycol (PEG) when subjected to melt extrusion undergoes 20% conversion to homogenously distributed C-dots with a diameter of 10–40 nm (Figure 12a). The ^1^H NMR spectrum of EA (Figure 12b) shows peaks at 3.7 and 2.8 ppm, corresponding to the CH_2_ protons of the –CH_2_OH and –CH_2_NH_2_ groups, respectively, while both PE/C-dot and PEG/C-dot nanocomposites display multiplets at 3.7 ppm but do not show peaks at 2.8 ppm, indicating the complete removal of EA. The C-dots exhibit QY from 3 to 11% and impart excitation wavelength-dependent fluorescence to the polymeric nanocomposites so that PE/C-dot (Figure 12c) and PEG/C-dot (Figure 12d) appear blue, green and red when illuminated with violet, blue and green light, respectively.

Lian et al. [65] reported the synthesis of stretchable and mechanically robust polyurethane (PU)/C-dots films by heating o-phenylenediamine (o-PD), m-phenylenediamine (m-PD) and 1,2,4-triphenylamine (1,2,4-3TD) dispersed within waterborne PU, thus resulting in blue, green and red nanocomposites, respectively. The *in situ*-generated C-dots with a size of 1.5–4 nm are well-dispersed within the polymer and remain embedded within the matrix when the composite is soaked on water for a prolonged period of time, due to the bonding between the carbonyl groups of PU and the amino groups of the C-dot precursors. Ahn et al. [66] demonstrated that glutathione and CA in the presence of ΤEMPO-oxidised cellulose nanofibre (CNF) when heated at 120 °C in a Teflon-lined autoclave produce *in situ*-generated C-dots with a diameter close to 3 nm that are connected to CNF via amide bonding. The CNF/C-dot membrane showed improved water flux and selective removal of cationic dyes (99.8% for methylene blue and 99% Janus Green B), thus showing great promise for water treatment applications.

*In situ*-generated C-dots have been shown to impart fluorescence characteristics to polymeric materials, but their potential to confer antimicrobial, antioxidant and photocatalytic properties has yet to be demonstrated. Likewise, while the *in situ* generation of CNT and graphene-based materials has been reported, the performance characteristics of the nanocomposites in terms of mechanical properties, electronic conductivity and antimicrobial behaviour remain to be investigated.

### 2.3. Titanium Dioxide and Silica-Based NPs

The *in situ* deposition of TiO_2_ NPs on polysulfone (PSF) ultrafiltration membranes proceeds via the interfacial polymerisation reaction of piperazine (PIP) and 1,3,5-benzenetricarbonyl trichloride (TMC) to generate an active PA layer and a subsequent hydrolysis reaction of tetra-butyl ortho-titanate (TBOT) [67]. The PSF/TiO_2_ membrane thus received showed improved water flux and a rejection of Na_2_SO_4_ and NaCl compared to its *ex situ*-prepared counterpart with identical TiO_2_ content.

Musto et al. [68] followed a sol-gel approach to prepare polyimide (PI)-silica hybrids by dispersing tetraethoxysilane (TEOS) in ethanol and aqueous HCl solution, and the hydrolysed alkoxylane solution was added dropwise to the polyamic acid (the PI precursor) solution. The mixture was spread on a glass plate and was left to dry before it was cured stepwise up to 300 °C, generating hybrids that showed simultaneous enhancements in fracture toughness, modulus and yield stress. Shen et al. [69] demonstrated the formation of PA/silica thin membranes via the *in situ* polymerisation of SiCl_4_ in the organic phase. A PSF ultrafiltration membrane was immersed in an aqueous solution containing m-phenylenediamine (MPD) and triethylamine (TEA) and was then placed in contact with a solution containing SiCl_4_, TMC and Isopar G. It was demonstrated that the incorporation of 0.02% SiCl_4_ improves the water permeability of the PA membrane by 171%, while the NaCl rejection remains essentially unaltered.

**Figure 13:**
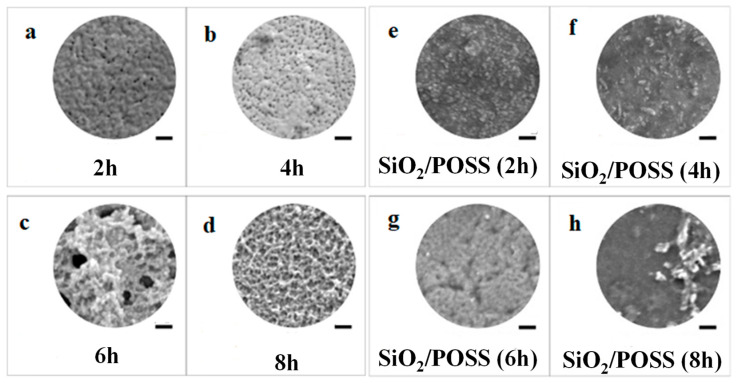
SEM images of: (**a**–**d**) POSS layer deposited of Kapton film at different growth times (2 h, 4 h, 6 h and 8 h), (**e**–**h**) sputter-deposited SiO_2_ coatings on different POSS/Kapton films. Scale bars represent 200 nm. Adapted with permission from Ref. [70].

A Kapton film was alkali-treated to generate carboxylate (–COO–) groups on its surface that subsequently were able to react with the amine groups of γ-aminopropyltriethoxysilane (APTES) in the presence of the tetraethylammonium hydroxide (TEAOH) catalyst, resulting in the *in situ* growth of polyhedral oligomeric silsesquioxane (POSS) NPs [69,70]. The SEM images of *in situ*-generated Kapton/POSS films at different growth times are shown in Figure 13a–d. It was noted that after 2 h of growth, the silanoxy groups (–Si–OC_2_H_5_) in the APTES molecules were partially hydrolyzed (–Si–OH), while after 6 h they were completely hydrolyzed (Si–O–Si), and the WCA dropped from 101° (observed for the original Kapton) to 71° and ultimately 30°. Subsequently, a SiO_2_ layer was deposited on the surface of the POSS layer by magnetron sputtering to prepare Kapton/POSS/SiO_2_ films. The SiO_2_ particles were easily aggregated on the partially hydrolysed organosilicon substrate (Figure 13e), and the pore structure was maintained in Figure 13f,g, but pore blockage and structural heterogeneities were observed in Figure 13h. Atomic oxygen radiation assessment indicated that the erosion yield of Kapton/POSS/SiO_2_(6 h growth) was dramatically improved to 0.19 × 10^−24^ cm^3^/atom compared to 3.19 × 10^−24^ cm^3^/atom of the original Kapton film.

The structure–properties relationships of those nanocomposites should be further established, and their adaptation and application in hydrogen storage, chemical sensing, mechanical reinforcement and corrosion prevention should be explored.

### 2.4. Layered Double Hydroxide (LDH) and Hectorite

The *in situ* growth of vertically aligned nanosheets of Mg-Al LDH on polyester fabric was reported by Aladpoosh et al. [71]. In short, Al(NO_3_)_3_ · 9H_2_O and Mg(NO_3_)_2_ · 6H_2_O were added to aqueous NaOH solution under vivid stirring to promote the formation of Mg-Al LDH seeds that were isolated via centrifugation and redispersed in water in which the polyester fabric was immersed and heated for 24 h at three different temperatures at 80, 100 and 120 °C, respectively. Well-formed nanosheets were observed only for the samples treated at 100 and 120 °C, and the burning behaviour of Mg-Al LDH-covered fabrics was found to monotonically improve with the treatment temperature. The formation of a compact and uniform nest-like Mg-Al LDH network on the fabric surface resulted in reduced air permeability. A similar process enabled the *in situ* construction of the 2D Mg-Al LDH coating through urea hydrolysis on a cotton fabric, while coating with stearic acid (SA) imparted superhydrophobicity, and the cotton/Mg-Al LDH/SA demonstrated oil/water separation efficiency better than 98% and showed superior stain resistance [72].

The *in situ* hydrothermal crystallisation of hectorite [73] takes place under reflux at 100 °C for 2 days of an aqueous gel comprising silica sol, Mg(OH)_2_, LiF and different polymer matrices. The polymeric matrices considered were poly(vinylpyrrolidone) (PVP), hydroxypropylmethylcellulose (HPMC), polyacrylonitrile (PACN), polydimethyldiallyl-ammonium chloride (PDDA) and polyaniline (PANI). All those polymers are positively charged, save for PDDA, and thus can penetrate the interlayer galleries of the hectorite, generating exfoliated structures for PACN/hectorite and PANI/hectorite, and intercalated structures for all other systems. The method yields nanocomposites with a maximum polymer content of 86%, 57%, 44%, 35% and 19% for PACN, PANI, HPMC, PVP and PDDA, respectively. Further work should focus on assessing the macroscopic behaviour of those materials and their use in highly demanding applications.

### 2.5. Lignin and Hydroxyapatite

A suspension of deprotonated, and thus negatively charged, lignin in acetone/water was mixed with positively charged chitin nanofibres (ChNF), and the strong electrostatic interactions facilitated lignin adsorption followed by nucleation and the growth of lignin NPs with a size of 46 ± 17 nm [74]. A similar process was followed using CNF, but due to electrostatic repulsions between CNF and lignin the distribution of the resultant lignin NPs was not homogenous, while their size was 28 ± 7 nm. Within the ChNF/lignin and CNF/lignin nanocomposites, the WCA increases as a function of NP loading, and their mechanical properties were not compromised compared to their uncoated counterparts. The ChNF/lignin nanocomposites show excellent UV blocking capabilities and high antioxidant activity, making them ideal candidates for food packaging application.

The *in situ* crystallisation of hydroxyapatite (HAp) in the presence of PVA took place via the slow addition of CaCl_2_ to a PVA aqueous dispersion, followed by the gradual addition of the NaH_2_PO_4_ solution, a process that produces a milky suspension at pH 10–11. Due to the nucleating efficacy of the side groups of PVA, the *in situ*-generated HAp crystals show less pronounced agglomeration compared to crystals prepared in the absence of PVA. The *in situ*-generated PVA/HAp nanocomposites showed superior mechanical toughness due to strong NP–matrix interactions compared to their *ex situ* counterparts at identical filler loading [75,76]. By virtue of its bio-adhesive nature, Ryu et al. [77] explored the use of PDA coating as a supporting layer for the growth of HA over a substrate material. A Ti substrate was submerged in a dopamine (DA) solution in tris buffer with pH = 8.5 to generate a thin PDA coating on its surface; it was then transferred to a simulated body fluid (SBF) containing vital ions such as Na^+^, K^+^, Ca^2+^, Mg^2+^, Cl^−^, HCO_3_^−^, SO_4_^2−^ and HPO_4_^2−^ in tris buffer at pH = 7.4 and was incubated at 37 °C to promote the biomimetic mineralisation of HA.

Overall, the *in situ* growth of lignin and hydroxyapatite NPs results in nanocomposites with superior mechanical and barrier properties suitable for advanced food packaging and biomedical applications.

### 2.6. In Situ NP Formation Simultaneously with Additional Major Structural Modifications

**Figure 14:**
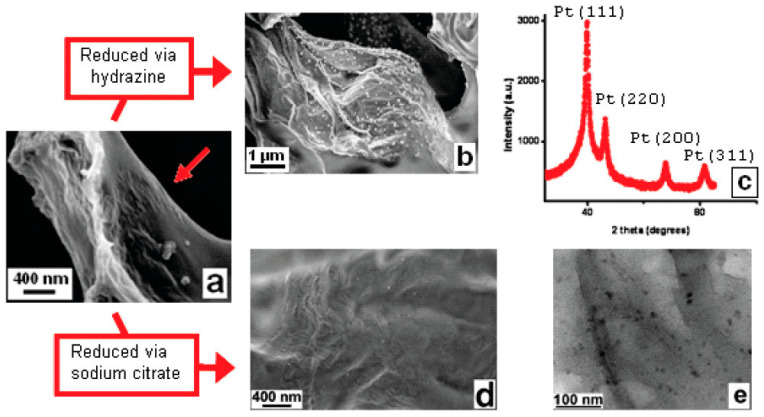
(**a**) SEM image of a freeze-cast Nafion/GO/H_2_PtCl_6_ microporous scaffold showing the presence of GO nanosheets (arrow) on the surface of the macropores. (**b**) Pt NPs deposited on the surface of graphene, after hydrazine treatment, and (**c**) X-ray diffraction (XRD) pattern of Pt NPs. (**d**,**e**) SEM and TEM images, respectively, of Pt NPs deposited on the surface of graphene, after treatment with sodium citrate. Adapted with permission from Ref. [78].

Estevez et al. [78] reported the preparation of multifunctional microporous scaffolds comprising Nafion, graphene and Pt NPs (Figure 14a). To that end, Nafion/GO/H_2_PtCl_6_ scaffold was prepared via ice-templating and was subsequently exposed to hydrazine to facilitate the dual *in situ* reduction in GO towards graphene platelets that adopt their characteristic crumpled appearance and H_2_PtCl_6_ towards Pt NPs with a size of 50–100 nm that were anchored on the surface of the graphene (Figure 14b). The diffraction peaks in Figure 14c correspond to the Pt (111), Pt (200), Pt (220) and Pt (311) reflections of the Pt fcc crystals. Following reduction with sodium citrate, smaller Pt NPs, with an average diameter close to 10 nm, are obtained (Figure 14d,e). Due to the reduction in the GO to the electrically conducting graphene, the resistance of the nanocomposite is decreased by at least four orders of magnitude (at low relative humidity).

**Figure 15:**
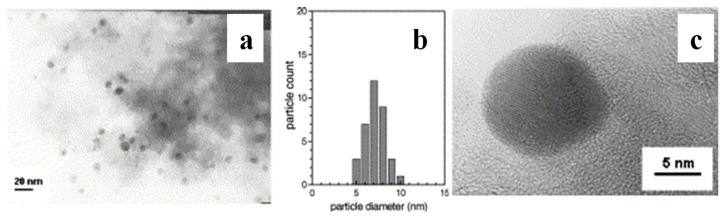
(**a**) TEM image, (**b**) size distribution and (**c**) HRTEM of *in situ*-generated PAN/Ag nanocomposite. Adapted with permission from Ref. [79].

Zhang et al. [79] reported that AgNO_3_ and benzoin methyl ether (photoinitiator) were dissolved in acrylonitrile (AN) monomer at room temperature, and the solution was subjected to UV radiation to simultaneously facilitate the polymerisation of polyacrylonitrile (PAN) and the reduction in Ag^+^ towards Ag NPs with a size of 5–10 nm that are well-dispersed within the PAN matrix (Figure 15).

**Figure 16:**
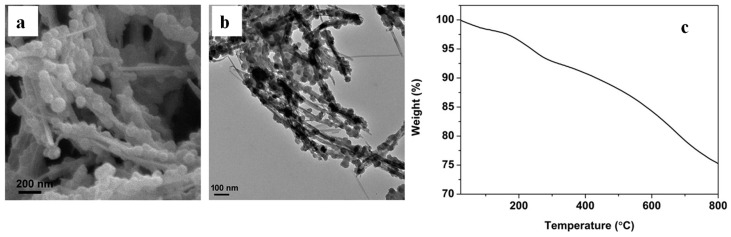
PPy/Ag/clay nanocomposites (mass ratio of ATP to pyrrole was 20:100): (**a**) SEM, (**b**) TEM images and (**c**) TGA. Adapted with permission from Ref. [80].

Nanocomposites comprising polypyrrole, silver and attapulgite (PPy/Ag/ATP) were prepared via the UV-induced polymerisation of pyrrole in the presence of silver nitrate that acted as a photoinitiator and silver source, while ATP clay acted as a templet to generate a beads-on-a-string morphology (Figure 16a,b). The reaction is completed in 10 min with a yield of 80%, and the nanocomposites show a unique combination of electrical, antibacterial and mechanical properties [80]. The Thermogravimetric Analysis (TGA) plot of PPy/Ag/ATP shown in Figure 16c is dominated by the evaporation of adsorbed water below 120 °C, while at higher temperatures the removal of coordination water and the degradation of PPy chains take place. Singh et al. [81] prepared PPy/Ag nanocomposite films on N-(3-trimethoxysilylpropyl)pyrrole-modified, biaxially oriented PET by photopolymerisation of pyrole in the presence of AgNO_3_ at exposure times varying from 15  to 180 min. The films thus prepared showed advanced H_2_S and NH_3_ gas-sensing properties.

**Figure 17:**
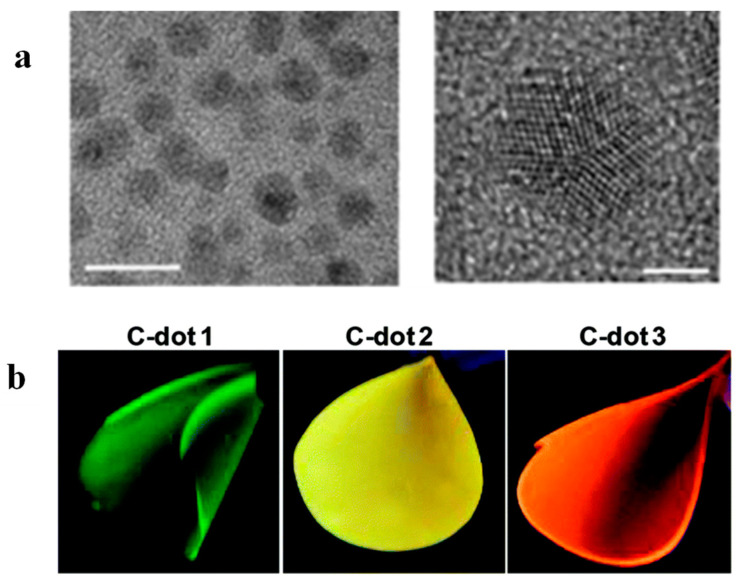
(**a**) TEM images of C-dots extracted from the PDMS/C-dot1 nanocomposite. The scale bars correspond to 10 nm (**left** image) and 2 nm (**right** image). (**b**) Photos of PDMS/C-dot1, PDMS/Cdot2 and PDMS/C-dot3 nanocomposites under illumination at 365 nm. The numbers refer to the C-dot precursors 1,2,3 corresponding to 6-O-(O-O′-Di-lauroyl-tartaryl)-d-glucose, 6-O-(O-O′-Di-lauroyl-tartaryl)-l-ascorbic acid and Vitamin B1 + oleic acid, respectively. Adapted with permission from Ref. [82].

Bhunia et al. [82] demonstrated the one-pot thermal synthesis of polydimethylsiloxane (PDMS) nanocomposites, where the embedded graphitic C-dots with a size of 4.2 ± 0.6 nm (Figure 17a) were prepared simultaneously with the build-up of the polymeric host. In particular, silicone elastomer base was mixed with a silicone elastomer curing agent, three different C-dot precursors were added, and the mixture was maintained at 75 °C for 1 h followed by heating at 127 °C for another 1.5 h. The precursors used were 6-O-(O-O′-Di-lauroyl-tartaryl)-d-glucose, 6-O-(O-O′-Di-lauroyl-tartaryl)-l-ascorbic acid, Vitamin B1 + oleic acid, yielding green PDMS/C-dots1, yellow PDMS/C-dots2 and orange PDMS/C-dots3, upon illumination at 365 nm as shown in Figure 17b.

In short, it is interesting to highlight the versatility and adaptability of this method in the sense that it can be flexibly incorporated into complex fabrication strategies for the built-up of multi-component and multi-functional materials.

## 3. Conclusions and Outlook

To the best of our knowledge, this is the first report that, together, presents the various systems prepared via the *in situ* growth of NPs on and within a polymeric material, and it demonstrates the inherent limitations and advantages of the method while pointing to areas with significant potential for further advances. Table 1 summarises representative contributions in this area. The preparation of nanocomposites on the basis of this method can be triggered via radiation, a thermal event or a chemical reaction and it, therefore, cannot be applied for polymers that are susceptible to those triggers. For example, thermoplastic polymers (PA6, PP, PLA, PE, PEG, etc.) are ideal matrices for thermally generated NPs, in contrast to thermosetting polymers that are not suitable for this purpose.

The approach results in well-dispersed systems with strong particle/host interface adhesion, thus ensuring longevity and minimizing NP detachment compared to their *ex situ* fabricated counterparts. So far, the method has been explored systematically to produce metallic (Ag, Au, Pt and Pd) nanocomposites for antimicrobial textiles and antifouling membranes with improved water flux, salt rejection and resistance to biofilm formation. Nevertheless, further studies are needed to assess the long-term performance of the membranes and any possible bioaccumulation effects.

A number of studies provide proof-of-concept demonstration for the fabrication of nanocomposites comprising *in situ*-generated carbon-based and silicon-based NPs, titania, hydroxyapatite, lignin, titanium oxide, layered double hydroxide and hectorite. The structure–properties relationships of those nanocomposites should be further established, and their applicability in hydrogen storage, chemical sensing, mechanical reinforcement and corrosion prevention should be examined in a systematic manner.

Particular interest lies in the *in situ* generation of Ag NPs and C-dots during melt extrusion, given that the method is thoroughly compatible with standard industrial procedures and gives rise to systems with interesting optical properties. Further studies in this area are needed to exploit the antimicrobial potential and the photocatalytic activity of the *in situ*-generated C-dots and their application in environmental remediation and light harvesting.

Finally, it should be noted that this method can be integrated in a multi-step strategy designed to deliver complex architectures and that the *in situ* formation of NPs can take place synchronously with other major structural modifications of the system.

## Figures and Tables

**Table 1:** *In situ*-generated NPs in polymer nanocomposites.

Types of Nanoparticles	Inherent Characteristics	References
Au, Ag, Pt, Pd, ZnO, Cu, Fe_2_O_3_ and Ce/Au	Antimicrobial Antifouling Catalytic activity Colourants	[26,27,28,29,30,31,32,33,34,35,36,37,38,39,40,41,42,43,44,45,46,47,48,49,50,51,52,53,54,55]
CNTs, graphene oxide	Absorption capacity (Mechanical reinforcement) (Electronic conductivity) (Barrier properties)	[59,60]
C-dots	Fluorescence (Antimicrobial) (Antioxidant)	[64,65,66]
TiO_2_	(Photocatalytic properties)	[67]
SiO_2_	Mechanical reinforcement	[68,69]
POSS	Erosion protection (Low dielectric response)	[70]
LDH	Barrier properties	[71,72]
Hectorite	(Mechanical reinforcement) (Barrier properties)	[73]
Lignin	UV blocking Antioxidant activity	[74]
Hydroxyapatite	Bioadhesion Mechanical reinforcement	[75,76,77]

In parentheses, expected performance characteristics not yet reported in the literature for nanocomposites bearing *in situ*-generated nanoparticles are listed.
